# Effect of Low-Pressurized Perfusion with Different Concentration of Elastase on the Aneurysm Formation Rate in the Abdominal Aortic Aneurysm Model in Rabbits

**DOI:** 10.1155/2016/6875731

**Published:** 2016-11-14

**Authors:** Maoxiao Nie, Yunfeng Yan, Xinhe Li, Tingting Feng, Xin Zhao, Mingduo Zhang, Quanming Zhao

**Affiliations:** Beijing Anzhen Hospital, Capital Medical University, Anzhen Road, Chaoyang District, Beijing 100029, China

## Abstract

Establishing an animal model of abdominal aortic aneurysm (AAA) is the key to study the pathogenesis and the pathophysiological features of AAAs. We investigated the effects of low-pressurized perfusion with different concentrations of elastase on aneurysm formation rate in the AAA model. Fifty male New Zealand white rabbits were randomly divided into A, B, C, D, and E groups. 10 *μ*L of normal saline was perfused into the abdominal aorta in group A and 1 U/mL, 10 U/mL, 100 U/mL, or 200 U/mL of elastase was, respectively, perfused for the other four groups. All the animals were perfused for 7 min. Doppler ultrasound examinations of the abdominal aorta were performed before surgery and on day 14 after surgery. The rabbits were sacrificed and the perfused segment of the abdominal aorta was observed visually and after staining. The aneurysm formation rate of group A, group B, group C, group D, and group E was, respectively, 0%, 0%, 33.3%, 102.5–146.8%, and 241.5–255.2%. The survival rate of five groups was 90%, 90%, 90%, 90%, and 40%, respectively. So, we concluded that low-pressurized perfusion with 100 U/mL of elastase can effectively establish AAAs in rabbits with a high aneurysm formation rate.

## 1. Introduction

Abdominal aortic aneurysm (AAA) is a common and severe cardiovascular disease, with an incidence of 5–8% in males older than 60 years of age [1]. However, once the AAA ruptures, the mortality rate can reach 80–90% [2]. The pathogenesis of AAAs is still unclear. By establishing an animal model of AAA, we can study the pathogenesis and the pathophysiological features of AAAs and determine measures for early prevention and treatment. Several small animal models have been developed to assist in understanding the mechanisms of AAA pathogenesis. Anidjar et al. [3] first introduced an elastase-induced AAA, one of the most commonly used models in rats. Periarterial elastase incubation is also used to create an aneurysm in the carotid [4–7] and aortic arteries [8–10] in small animals. Currently, elastase perfusion is the most commonly used method for animal models of AAA. However, as shown by the existing literature, different production methods of abdominal aortic aneurysm model, the elastase concentrations vary greatly [11–14]. To evaluate the effect of elastase concentrations, we established an AAA model in rabbits by perfusing different concentrations of elastase. The aneurysm formation rate and dilation of the abdominal aorta were measured. Thus, the optimal concentration of elastase for inducing an AAA was determined.

## 2. Materials and Methods

### 2.1. Materials

Fifty healthy male New Zealand white rabbits (Beijing Haidian Xingwan Animal Breeding Farm, License no. SCXK (Beijing) 2006-0006), weighing approximately 2.50 ± 0.20 kg, were used. The rabbits were allowed free access to water and food and were reared in separate cages with an ambient temperature of 23–25°C. The housing environment was dry with good ventilation, adequate lighting, and easy drainage. The cage was 60 cm in width and 50 cm in height, and one animal was fed per cage. The feed formulation included alfalfa powder, wheat bran, flour, corn, soybean meal, fish meal, bone meal, yeast powder, salt, and fish liver oil. Clean tap water was provided as drinking water. Pancreatopeptidase E (elastase at PH 8.8, 37.0°C) (45124, Sigma-Aldrich, St. Louis, Missouri, USA) was diluted to concentrations of 1 U/mL, 10 U/mL, 100 U/mL, and 200 U/mL. Sodium chloride (0.9%) was purchased from Shandong Hualu Pharmaceutical Co., Ltd. A 4 F Fogarty double-lumen balloon catheter was purchased from Beijing Life Green Technology Co., Ltd. The hematoxylin and eosin (HE) stain kit, elastic Van Gieson (EVG) stain kit, CD68 stain kit, and Masson's stain kit were purchased from Wuhan Guge Biotechnology Co., Ltd. Pentobarbital sodium was used for anesthesia, and the heparin sodium used for injection was purchased from Beijing Double-Crane Pharmaceutical Equipment Co, Ltd.

### 2.2. Methods

This study was performed strictly according to the recommendations in the Guide for the Care and Use of Laboratory Animals of the National Institutes of Health. The protocol was approved by the Committee on the Ethics of Animal Experiments of the Anzhen Hospital.

#### 2.2.1. Grouping

Fifty male New Zealand white rabbits were randomly divided into five groups (groups A, B, C, D, and E), with 10 rabbits in each group. Group A was perfused with 10 *μ*L of normal saline using a double-lumen catheter; group B was perfused with 10 *μ*L of 1 U/mL elastase; group C was perfused with 10 *μ*L of 10 U/mL elastase; group D was perfused with 10 *μ*L of 100 U/mL elastase; and group E was perfused with 10 *μ*L of 200 U/mL elastase.

#### 2.2.2. Modeling

(1) Preparation: in order to prevent airway obstruction caused by vomiting during the operation, all the rabbits were fasted for at least 10 h and not allowed access to water 4 h before surgery. Intravenous injections of heparin sodium (125 U/kg) were performed 30 min before surgery, followed by anesthesia via an intraperitoneal injection of 3% pentobarbital sodium. The rabbits were shaved and disinfected, and draping was performed conventionally under sterile conditions. (2) Femoral artery dissociation: an incision was made at the right common femoral artery to separate and dissociate the femoral artery for approximately 0.5–1 cm. Threading was performed at the proximal and distal end. (3) Abdominal surgery: a midline incision was made to expose the abdominal aorta. One segment of the abdominal aorta that contained fewer branches was dissociated for approximately 1.5–2.0 cm (smaller branches were ligated using 0 silk thread). Rubber filter thread was placed, and caution was taken not to injure the lumbar artery. Threading was performed at the proximal and distal ends. (4) Puncturing and catheterization of the femoral artery: a small incision was made in the femoral artery, and a catheter was inserted. The proximal end of the femoral artery was relaxed, and the catheter was gently delivered into the dissociated segment of the aorta. The proximal end of the abdominal aorta was obstructed using an artery clip and rubber filter thread; the distal end was obstructed using a rubber filter thread. Thus, a closed lumen connected to the catheter was formed. Blood was withdrawn from the lumen, and normal saline was used to flush the lumen three times. (5) For different groups, the abdominal aorta was flushed with normal saline or 0.1 mL of different concentrations of elastase using the double-lumen catheter. The perfusion was not pressurized, and no limit was placed on the degree of vascular dilation. The perfusion fluid was removed after 7 min. Normal saline containing heparin was injected via the secondary lumen and withdrawn via the primary lumen. The abdominal aorta was washed repeatedly. (6) Restoration of blood flow through the abdominal aorta: the thread and the microcatheter were removed. The proximal end of the femoral artery was ligated, and the abdomen was closed. (7) Measures to prevent infection: penicillin (800,000 U) was injected intraperitoneally in each rabbit for 3 consecutive days after surgery. Animals were given a subcutaneous injection of buprenorphine hydrochloride (0.03 mg/kg, Drug Research Pharma, Tianjin, China) once a day for 3 days after the operation for analgesia. The blood pressure was monitored every day for 3 days after the operation, and the diet, drinking water, and activity status of the animals were observed. For any rabbits that showed clinical signs of illness or injury following surgery, the following measures were taken: (1) monitoring the vital signs of the animals; (2) observing the presence of bleeding, infection, infiltration, and other changes at the surgical site of the animal; and (3) prolonging antibiotic treatment time.

#### 2.2.3. Doppler Ultrasound Examination of the Abdominal Aorta before Surgery and on Day 14 after Surgery

Ultrasound examinations were performed in the coronal and transverse planes. Three positions of the aneurysm were selected in the transverse plane to calculate the maximum aneurysm diameter. The maximum lumen diameter was calculated using postprocessing software. The average value was recorded as the final result. The abdominal aorta dilation rate was calculated using the following formula: dilation rate = (inner diameter of abdominal aorta aneurysm−average inner diameter of abdominal aorta aneurysm)/average inner diameter of abdominal aorta; average inner diameter of an abdominal aorta aneurysm = (inner diameter of the upper part of aneurysm−inner diameter of the lower part of aneurysm)/2.

#### 2.2.4. Anesthesia Was Given, and a Laparotomy Was Performed on Day 14 after Surgery

The perfused segment of the abdominal aorta was harvested, and the rabbits were sacrificed by arterial air embolism. The air embolism euthanasia procedure was performed under sodium pentobarbital anesthesia, and all efforts were made to minimize suffering. The tissues were cut into 5 *μ*m sections and detected by HE, EVG, and Masson's staining.

#### 2.2.5. Statistical Analysis

The statistical analysis was performed with SPSS 17.0 software. All data were expressed as the means ± standard deviation. The data were analysed using one-way analysis of variance (ANOVA) or repeated measures ANOVA followed by Bonferroni post hoc tests. Differences were considered statistically significant at *P* < 0.05.

## 3. Results

Each group of A, B, C, and D had a rabbit died during the surgical procedures (one rabbit died due to aortic dissection during operation and other three died of vascular rupture during operation). Fourteen days after operation, nine rabbits survived in each group of A, B, C, and D, resulting in a survival rate of 90%. No rabbit died in group E during surgical procedures. In group E, six rabbits died within 1 week after surgery (animals were euthanized when they showed certain clinical signs indicative of illness/injury/morbidity) and 4 rabbits survived; the survival rate was 40%.

### 3.1. Visual Observation of the AAA (Figure 1)

Part of the blood vessels at the surgical site had no obvious expansion in groups A and B; group C showed slight expansion; compared with group A, groups D and E obviously expanded at the surgical site; the maximum rate of expansion was more than 100%.

### 3.2. Changes in the Diameter of the Abdominal Aorta upon Ultrasound Examination

**Figure 2:**
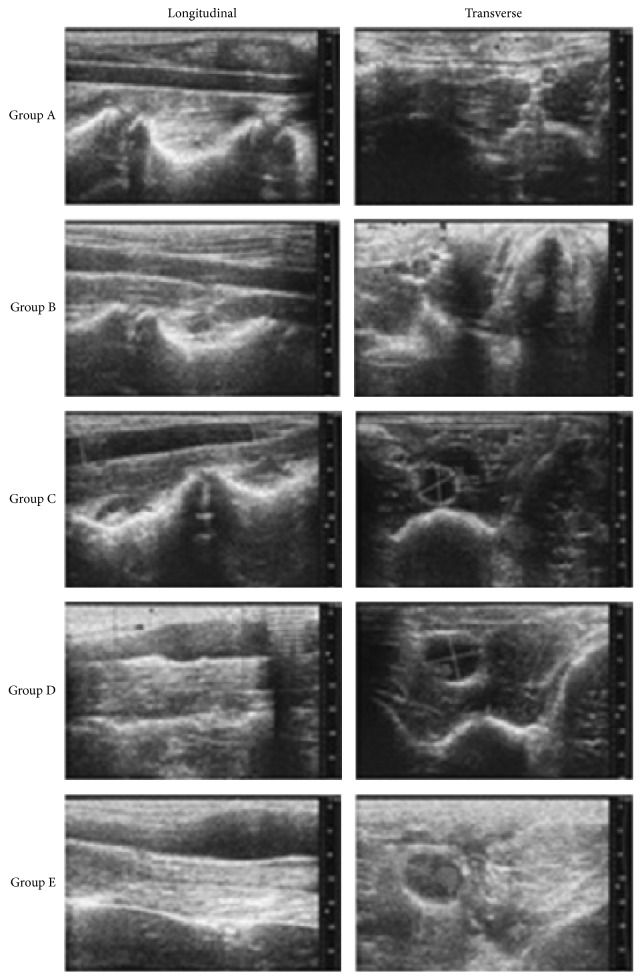
The diameter of the abdominal aorta differentially changed in response to different concentrations of elastase, as shown by Doppler ultrasound examinations of the abdominal aorta. The aneurysm diameter increased with an increasing concentration of elastase.

The perfused segment of the abdominal aorta did not show local enlargement after surgery in group A, and no aneurysm was formed. In group B, the maximum aneurysm diameter in the perfused segment was slightly increased compared to that before surgery; the local dilation rate was 21.4–29.7%, which did not satisfy the criterion of aneurysm formation, and the aneurysm formation rate was 0%. In group C, the local dilation rate was 31.2–55.3%, and 3 rabbits showed a dilation rate above 50%, which satisfied the criterion of aneurysm formation in the abdominal aorta. Therefore, the aneurysm formation rate of group C was 33.3%. In group D, the perfusion segment dilation rate was 102.5–146.8%, and the aneurysm formation rate was 100%; thus, the aneurysm formation rate was 100%. In group E, the local dilation rate was 241.5–255.2%, and the aneurysm formation rate was 100%. The results are shown in Table 1 and Figure 2.

### 3.3. Histological Changes Are Shown in Figure 3

**Figure 3:**
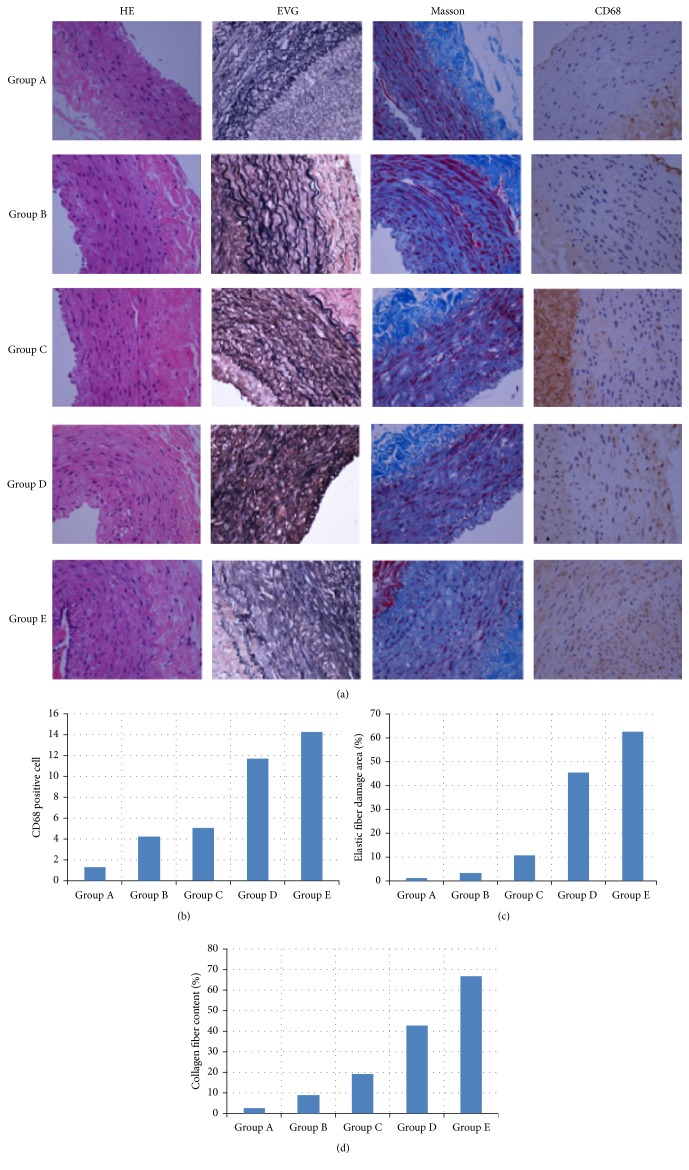
Histomorphometric changes in groups A to E (a). HE: hematoxylin and eosin stain (*∗*400); EVG: elastic Van Gieson stain (*∗*400), MASSON: Masson's stain (*∗*400). CD68: CD68 macrophages staining (*∗*400). As shown by CD68 staining, the number of CD68 positive cells infiltrating the aortic wall increased from group A to group E. Compared with group A, the number of CD68 positive cells in group D and group E increased significantly, *P* < 0.05; the difference was statistically significant (b). EVG stain shows that the elastic fibrous tissue wavy in group A is clear, while that in group E is in disorder and fuzzy. Compared with group A, the destruction amount of elastic fiber in group D and group E obviously increased, *P* < 0.05; the difference was statistically significant (c). Masson's stain shows that the collagen fiber content increased gradually from group A and group E. Compared with group A, the collagen fiber content in group D and group E increased significantly, *P* < 0.05; the difference was statistically significant (d).

HE staining observed the vascular wall structure. As the figure shows, the vascular wall of group A is structurally complete, and the membrane smooth muscle is ranged neatly, without inflammatory cell infiltration. While the structure and the integrity of aneurysm wall from group E are poor and the smooth muscle in the membrane is disorderly arranged, with infiltrating inflammatory cells in vascular wall, CD68 positive cells can further reflect the infiltrating degree of the inflammatory cells in aneurysm wall. As the figure shows, the number of CD68 positive cells from group A to group E aneurysm wall increased significantly. The number of CD68 positive cells in each group is, respectively, as follows: group A 1.32 ± 0.47; group B 4.24 ± 0.39; group C 5.07 ± 0.21; group D 11.72 ± 0.41; group E 14.29 ± 0.23. Compared with group A, the number of CD68 positive cells in group D and group E increased significantly, *P* < 0.05; the difference was statistically significant (Figure 3(b)); EVG staining observed the destruction degree of elastic fiber tissue in each group. From the figure, we can see that the elastic fibrous tissue wavy in group A is clear, while that in group E is in disorder and fuzzy. The damage area percentage of elastic fiber to total observation area is, respectively, as follows: group A 1.10%  ± 0.12%, group B 3.11%  ± 0.21%, group C 10.61%  ± 0.33%, group D 45.30%  ± 0.27%, and group E 62.64%  ± 0.45%; compared with group A, the destruction amount of elastic fiber in group D and group E obviously increased, *P* < 0.05; the difference was statistically significant (Figure 3(c)); Mason staining observed the changes of abdominal aortic aneurysm wall collagen fiber content in different groups. As the figure shows, the collagen fiber content increased gradually from group A and group E. The percentage of each collagen fiber content of total observation area is, respectively as follows: group A 2.62%  ± 0.11%, group B 8.90%  ± 0.19%, group C 19.11%  ± 0.20%, group D 42.78%  ± 0.23%, and group E 66.94%  ± 0.31%. Compared with group A, the collagen fiber content in group D and group E increased significantly, *P* < 0.05; the difference was statistically significant (Figure 3(d)).

## 4. Discussion

Abdominal aortic aneurysm (AAA), defined as a permanent localized aortic dilation with a diameter of 1.5 times the normal aorta diameter, is a silent degenerative disease that can be life-threatening [15]. The native AAA model induced by elastase perfusion is a standard aneurysm model for experimental research in rodents [1, 3, 16–21] and rabbits [22, 23]. Although the pathogenesis of AAA is still unknown, it is generally believed that the effects of elastase stimulated an elastolytic and inflammatory response in the aortic walls [24] and resulted in the continual progress of aneurysm development. In our study, we successfully developed an AAA model in rabbits by low-pressurized perfusion of 100 IU/mL elastase. We found that the aortic diameter increased with increasing elastase concentration. Lower concentration of elastase was unable to reliably induce AAA formation.

Histologically, the aneurysms are characterized by the near total destruction of the elastin matrix of the media. Busuttil et al. [25] had reported that elastase activity in the aneurysm wall was greater in intima media than in outer layers. White et al. [26, 27] had suggested that macrophages within the aortic media may be responsible for elastase secretion and subsequent aneurismal degeneration. They believed that medial injury with elastolysis was necessary for aneurysm formation, and the inflammatory response facilitated this process. The histological results of our study were coincidence with their. As shown by CD68 staining, the number of CD68 positive cells in the aortic wall increased from group A to group E. The more the positive cells, the more severe the inflammatory reaction whereas the less the positive cells, the less severe the inflammatory reaction. EVG staining showed the damage of the elastic fibers in the abdominal aortic wall. We can see that the elastic fibers of group A presented a wave-like texture without obvious damage. However, the texture of the elastic fibers in group E was obscured with residual elastic fibers. Compared to groups B, C, and D, group E showed the most severe damage to elastic fibers. Mason staining observed the changes of abdominal aortic aneurysm wall collagen fiber content in different groups. In our study, the collagen fiber content increased gradually from group A and group E. Compared with group A, the collagen fiber content in group D and group E increased significantly.

Our data suggested that the 100 U/mL group had the highest AAA formation rate and survival rate in the five groups with different perfusion concentrations. Azuma Junya and his colleagues reported that they had established a rat abdominal aortic aneurysm model by aorta perfusion, and the perfusion concentration of porcine pancreatic elastic protease was 1.5 U/mL. Bi et al. [14] reported that they had observed the effects of different concentrations of elastin by using outer periphery embedding method to establish rabbit AAA model of abdominal aortic aneurysm. They divided rabbits into five groups with the concentration of porcine pancreatic proteases 0, 0.1, 0.5, 1, and 10 U/*μ*L, respectively. Under the administration of perfusion pressure of 350 mmHg and perfusion concentration of 100 U/mL, Kobayashi [28] and his colleagues successfully established rabbit abdominal aortic aneurysm by using porcine pancreatic elastase. In our study, we used the same infusion concentration, but the perfusion pressure is much lower.

Low-pressurized perfusion was performed in our study. At present, in order to cause injury of aortic wall, most of the researches give perfusion pressure 300–400 mmHg in the process of making animal abdominal aortic aneurysm. Yamaguchi et al. successively established rabbit AAA through giving peak pressure between 300 and 400 mmHg in the isolated segment [29]. In our preliminary experiments, we found that high-pressurized perfusion (300 to 400 mmHg) might lead to entry of elastase into the blood circulation via the vessel wall microcirculation. This could increase the risk of acute pancreatitis and damage to other organs. Moreover, under high pressure elastase might leak outside of the lumen, leading to damage to the entire luminal layers and surrounding tissues. In our experiments, we only gave 100 mmHg intraluminal perfusion pressure. We found that low-pressurized perfusion not only avoided hemorrhaging but also successfully induced an AAA.

Different experiments take different perfusion time in order to establish AAA models; the perfusion time varies from 20 min to 120 min [3, 13, 17, 28]. Anidjar et al. firstly established a rat AAA by perfusing elastase for 20 min. In Yamaguchi et al.'s study, 76 male Sprague Dawley rats were infused with a solution of elastase for 10, 20, 30, 60, or 120 min or with saline for 30 min. They concluded that it was possible to shorten the elastase infusion time from 120 min to 30 min in the elastase-induced rat aneurysm model and shortening of infusion time could reduce the experimental time and mortality rate [30]. Shortening the perfusion time was also considerable in our study; compared with them, we found that l00 U/mL concentration of elastase perfused for only 7 min was able to induce AAA formation, and that was favorable for increasing the survival rate of animals. Carsten et al. [31] indicated that a longer perfusion time resulted in longer obstruction of the abdominal aorta and prolonged ischemia of the organs below the level of the abdominal aorta and that would lower the survival rate of animals and even cause paralysis of the hind limb. Another research confirmed that 71% of the rabbits have been shown to develop paraplegia after temporary occlusion of the infrarenal aorta for 20 min [32]. One hour of ischemia had produced spinal cord infarction in all of the animals examined [1]. Thus, by reducing the perfusion time, we have greatly reduced the overall operation time and the death rate.

Ding et al. [33] reported that elastase-induced saccular aneurysm in carotid artery maintained stability and patency during 2- and 5-year follow-up. However, some research showed that AAA model induced by chemical substance injury, such as elastase, calcium chloride, or papain, might not result in progressive enlargement. Origuchi et al. [34] reported that aneurysms induced by adventitial elastolysis did not progress, gradually shrank over 42 days, and healed spontaneously by 90 days. Bi et al. [35] reported that aneurysms showed progression after day 5; however, long-term follow-up to study the further changes of the model was unwarranted, considering that there was no evidence of aneurysm stability beyond 21 days in the present experiments. In our preliminary experiment, we observed that rabbit AAA developed immediately after operation in the elastase-induced model. This fast-injury model enlarged within 10–20 days and aneurysm shrank thereafter. That is the reason why we choose to kill animals 14 days after the operation.

Anidjar et al. first introduced a method to create an AAA model in rats. It is quite obvious that rat AAA model induced by this method was too small to perform further research. In our research, we present an easy, efficient, and reproducible way to create rapid dilation of rabbit aortic arteries to form a model of AAA. This simpler aneurysm model will be more valuable for elucidating AAA mechanisms and therapeutic interventions. We believe that our rabbit aneurysms models are more suitable for the future research.

## Figures and Tables

**Figure 1:**
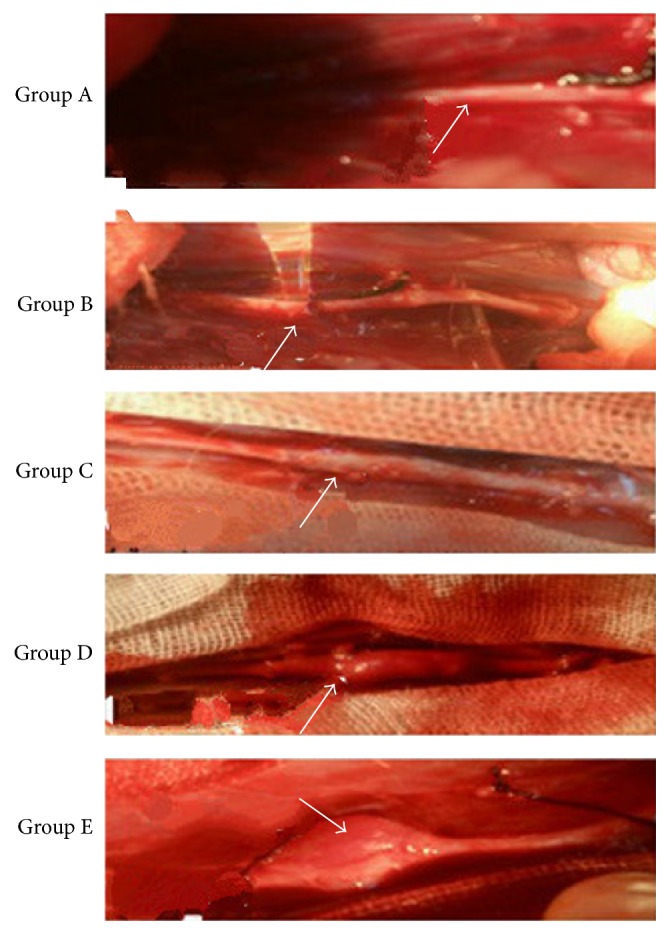
A segment of abdominal aorta photographed 14 days after surgery.

**Table 1:** Diameter changes measured by Doppler ultrasound.

	Maximum aneurysm diameter before surgery (mm)	Maximum aneurysm diameter after surgery (mm)	Dilation rate (%)	Aneurysm formation rate (%)	Survival rate
Group A	2.32 ± 0.03	2.32 ± 0.03	0	0/9 (0)	90
Group B	2.36 ± 0.05	2.95 ± 0.06	24.9 ± 2.5	0/9 (0)	90
Group C	2.39 ± 0.03	3.43 ± 0.17	43.9 ± 7.69	3/9 (33.3)	90
Group D	2.36 ± 0.04	5.32 ± 0.34	125.4 ± 16.1^*∗*^	9/9 (100)^*^	90
Group E	2.33 ± 0.03	8.11 ± 0.11	248.2 ± 5.6	4/4 (100)	40^⋆^

^*∗*^ *P* < 0.05, compared to groups A, B, and C; ^⋆^ *P* < 0.05, compared to groups A, B, C, and D.
